# NRP1 and GFAP Expression in the Medulloblastoma Microenvironment: Implications for Angiogenesis and Tumor Progression

**DOI:** 10.3390/cancers17152417

**Published:** 2025-07-22

**Authors:** Margarita Belem Santana-Bejarano, María Paulina Reyes-Mata, José de Jesús Guerrero-García, Daniel Ortuño-Sahagún, Marisol Godínez-Rubí

**Affiliations:** 1 Laboratorio de Patología Diagnóstica e Inmunohistoquímica, Centro de Investigación y Diagnóstico en Patología, Departamento de Microbiología y Patología, Centro Universitario de Ciencias de la Salud (CUCS), Universidad de Guadalajara, Guadalajara 44340, Jalisco, Mexico; santana.bejarano@gmail.com; 2 Doctorado en Ciencias en Biología Molecular en Medicina, Centro Universitario de Ciencias de la Salud (CUCS), Universidad de Guadalajara, Guadalajara 44340, Jalisco, Mexico; 3 Departamento de Disciplinas Filosófico, Metodológicas e Instrumentales, Centro Universitario de Ciencias de la Salud (CUCS), Universidad de Guadalajara, Guadalajara 44340, Jalisco, Mexico; paulina.reyes@academicos.udg.mx; 4 Banco de Sangre Central, Unidad Médica de Alta Especialidad (UMAE), Hospital de Especialidades (HE), Centro Médico Nacional de Occidente (CMNO), Instituto Mexicano del Seguro Social (IMSS), Guadalajara 44340, Jalisco, Mexico; jose.guerrero9683@academicos.udg.mx; 5 Departamento de Farmacobiología, Centro Universitario de Ciencias Exactas e Ingenierías (CUCEI), Universidad de Guadalajara, Guadalajara 44340, Jalisco, Mexico; 6 Laboratorio de Neuroinmunobiología Molecular, Instituto de Neurociencias Translacionales, Centro Universitario de Ciencias de la Salud (CUCS), Universidad de Guadalajara, Guadalajara 44340, Jalisco, Mexico

**Keywords:** medulloblastoma, tumor microenvironment, GFAP, CD68, astrocytes, tumor-associated microglia/macrophages (TAMs), neuropilin-1

## Abstract

**Background/Objectives**: Medulloblastoma (MB) is the second leading cause of cancer-related death in children. Its tumor microenvironment (TME) includes endothelial, glial, and immune cells that influence tumor architecture and progression. Neuropilin-1 (NRP1), a co-receptor for semaphorins and vascular endothelial growth factor (VEGF), is expressed in various cell types during oncogenesis, yet its role in MB progression remains unclear. This study aimed to evaluate the expression and localization of NRP1 and glial fibrillary acidic protein (GFAP) in MB tissue. **Methods**: We analyzed MB tissue samples using immunohistochemistry, immunofluorescence, and quantitative PCR. Samples were stratified by molecular subgroup (WNT, SHH, non-WNT/non-SHH). We assessed NRP1 expression in tumor-associated microglia/macrophages (TAMs) and endothelial cells, as well as GFAP expression in astrocytes and tumor cells. Histopathological correlations and survival analyses were also conducted. **Results**: NRP1 was consistently expressed by TAMs across all MB molecular subgroups. Tumor vasculature showed strong endothelial NRP1 expression, while perivascular astrocytic coverage was frequently absent. Astrocytic processes exhibited spatial differences according to tumor histology. In SHH-MBs, a subset of tumor cells showed aberrant GFAP expression, which correlated with tumor recurrence or progression. **Conclusions**: NRP1 and GFAP display distinct expression patterns within the MB microenvironment, reflecting subgroup-specific biological behavior. Endothelial NRP1 positivity combined with limited vascular-astrocytic interaction and aberrant GFAP expression in SHH-MB may contribute to dysregulated angiogenesis and tumor progression. These findings warrant further investigation to explore their prognostic and therapeutic implications.

## Simple Summary

Medulloblastoma is a type of brain tumor that mainly affects children and often has serious outcomes. In this study, we examined how certain proteins—neuropilin-1 and glial fibrillary acidic protein (GFAP)—are distributed in tumor tissue and how they relate to blood vessels, immune cells, and tumor behavior. By studying different types of medulloblastoma, we found that these proteins show specific patterns depending on the tumor subtype. Our results suggest that abnormal blood vessel formation and the presence of certain cell markers may be linked to tumor growth and recurrence. These findings help us better understand how the tumor develops and may eventually lead to new ways to predict disease progression or create targeted treatments.

## 1. Introduction

Medulloblastoma (MB) is the most common solid malignant tumor of the central nervous system (CNS) in children, accounting for 69.5% of embryonal CNS tumors in individuals under 20 years of age [1]. This World Health Organization (WHO) grade IV neoplasm arises from impaired developmental processes affecting cerebellar and brainstem precursor cells during human embryogenesis, involving activation of the WNT and Sonic Hedgehog (SHH) signaling pathways and amplification of *MYC* and *MYCN* oncogenes [2]. These molecular alterations define four distinct subgroups: MB-WNT, MB-SHH, and MB-No WNT/No SHH (subgroups 3 and 4), each with different origins, demographic characteristics, and clinical outcomes [3,4]. In addition, MB can be categorized into four morphological patterns: MB classic (MBC), MB desmoplastic/nodular (MBDN), MB extensive nodular (MBEN), and MB large cell/anaplastic (MBLCA) [2].

Reported evidence underscores the crucial role of the tumor microenvironment (TME) in the biology of MB, which influences tumor progression, survival, treatment resistance, and recurrence [5,6]. The TME comprises various non-neoplastic cell types, including endothelial cells, neurons, astrocytes (glial fibrillary acidic protein, GFAP+), and tumor-associated microglia/macrophages (TAMs, CD68+), which dynamically interact with tumor cells to support angiogenesis, invasion, and immunomodulation [7,8,9]. TAMs within the TME can exhibit either pro-inflammatory (M1) or anti-inflammatory (M2) polarization. M2-like TAMs are associated with immunosuppression and tumor progression, while M1-polarized TAMs promote anti-tumor responses [5,10]. MB-SHH tumors contain the highest number of TAMs that have variable effect on tumor growth [8,11,12,13,14].

Astrocytes, traditionally considered homeostatic regulators in the CNS, also play an active role in tumor dynamics and are involved in neurotransmitter recycling, ion buffering, and maintenance of the blood–brain barrier (BBB) [15]. They express components of the SHH signaling pathway such as PTCH1 and GLI [15,16,17] and can secrete factors such as CD133 that increase the invasive potential of MB stem-like cells [18]. SHH ligands secreted by tumor cells can activate astrocytes and promote local tumor-supportive dynamics [19]. Interestingly, GFAP expression has also been observed in MB tumor cells themselves, particularly in the SHH subgroup, and is associated with heterogeneous clinical outcomes [14,20,21].

Neuropilin-1 (NRP1), a type I transmembrane glycoprotein, has emerged as a key player in tumor biology, with roles in angiogenesis and tumor progression through vascular endothelial growth factor (VEGF), transforming growth factor-beta (TGF-β), and placental growth factor (PlGF) signaling pathways [13,22,23]. NRP1 is involved in axon guidance [24] and is widely expressed during development [25,26]. In MB, NRP1 promotes tumor cell survival and supports the maintenance of cancer stem cells, metastasis and angiogenesis [13,27,28]. In other cancers, NRP1 modulates the polarization of TAMs towards pro-tumoral phenotype under hypoxia and contributes to immune evasion [29,30]. Furthermore, NRP1 is expressed in endothelial and astrocytic cells, where it can influence both BBB stability and angiogenesis [31,32].

Angiogenesis in medulloblastoma exhibits a subgroup-specific molecular pattern that is strongly associated with tumor behavior and disease progression. Due to downregulation of BBB genes and loss of endothelial tight junction integrity, MB-WNT exhibits a densely and abnormally branched permeable vasculature that facilitates intratumoral accumulation of chemotherapeutic agents and improves the prognosis of the subgroups. In comparison, MB-SHH tends to have an intact BBB with low vascular density and well-organized vascular arrangement [33]. However, adult SHH-MBII tumors exhibit high VEGFA expression, which correlates with increased angiogenic activity and poorer survival, suggesting heterogeneity within the SHH subgroup [34]. Furthermore, molecular studies in mice show that the PIGF/NRP1 pathway promotes angiogenesis and tumor aggressiveness in all subtypes, with NRP1 expression being associated with poor prognosis [13]. According to Thompson et al. (2017), Group 3 tumors also exhibit aggressive clinical behavior, increased VEGFA levels, and diverse vascular organization [35]. Despite these advances, current studies have primarily focused on the endothelial and pericytic components of MB-BBB, ignoring the presence, distribution, and properties of astrocytes as critical components of gliovascular interactions in intratumoral vessels and their relationship to NRP1 expression in tumor vessels and TAM cells.

Given these gaps in our current knowledge, a better understanding of NRP1 expression in the tumor microenvironment is needed. While the expression patterns and functional effects of NRP1 in non-neoplastic cells of the MB microenvironment remain poorly characterized, mapping the spatial and cellular distribution of NRP1 in tumor vasculature and glial cells may shed light on its role in tumor–microenvironment interactions. Therefore, in this study, we analyzed the expression patterns of NRP1 and glial markers in MB tissue and evaluated their associations with tumor morphology, molecular subgroup, and clinical behavior.

## 2. Materials and Methods

### 2.1. Tissue from Medulloblastoma Patients

The present study was conducted in accordance with the principles of the Declaration of Helsinki and received approval from the Ethics Committee of the Centro Medico Nacional del Occidente de México, Hospital Civil Juan I. Menchaca, and the Universidad de Guadalajara, Jalisco, Mexico (approval numbers R-2021-1301-123, 00123, and CI-02021, respectively). The 45 paraffin-embedded tissues originated from pathologic autopsies and neurosurgical procedures performed between 2005 and 2022.

### 2.2. Automated Immunohistochemistry (IHC)

All formalin-fixed paraffin-embedded (FFPE) tissue samples (n = 45) were cut into 3 µm thick slices, which were then immunostained using an automated BOND equipment (Leica Biosystems, Deer Park, IL, USA). Immunodetection was performed according to the guidelines of the manufacturer of the Bond polymer refine detection system (DS9800, Leica Biosystems, Deer Park, IL, USA). Antigen recovery was performed with citrate buffer solution (pH 6.0) or EDTA buffer solution (pH 8.0). The primary antibodies anti-NRP1 (1:250, Ab81321, Abcam, Cambridge, UK), anti-CD68 (1:100, CM033A, Biocare Medical, LLC, Pacheco, CA, USA), and anti-GFAP (1:250, 258R-15, Cell marque, Rocklin, CA, USA) were incubated for 30 min. The cecal appendix served as a positive control tissue for NRP1 and CD68; the cerebellum was used as a positive control tissue for GFAP. The tissues without primary antibodies were used as negative controls (Supplementary Figure S1).

### 2.3. Quantification of Protein Staining

Images were acquired using the Aperio LV1 (Leica Biosystem Imaging Inc., Deer Park, IL, USA). Ten different areas of each sample were analyzed to obtain a percentage of positive cells, and 1 mm^2^ was used to calculate the density of positive cells or vessel luminal areas in each sample using Qupath (open-source software for digital image analysis in pathology, V.0.3.0) (Bankhead et al., 2017 [36]). Positive staining (3,3′-diaminobenzidine staining) was counted using hematoxylin detection as a reference measure (Supplementary Figure S2).

### 2.4. Immunofluorescence Technique (IF)

Slides with formalin-fixed paraffin-embedded MB tissue (n = 15) were deparaffinized and rehydrated. Antigen recovery was performed with citrate buffer solution (pH 6.0) or EDTA buffer solution (pH 8.0). NovacastraTM (Leica Biosystems, Deer Park, IL, USA) protein block was used for 30 min to reduce non-specific binding. The primary antibodies anti-NRP1 (1:250, Ab81321, Abcam, Cambridge, UK) and anti-CD68 (1:100, CM033A, Biocare Medical, LLC, Pacheco, CA, USA) were incubated overnight at 4 °C. Tissues were treated for 30 min with fluorophore-conjugated secondary antibodies (mouse anti-rabbit, IgG-CFL 488, sc-516248; m-IgG Fc 594, sc-5333655, Santa Cruz Biotechnology, Inc., Dallas, TX, USA). Sections were counterstained with 4′,6-diamidino-2-phenylindole (DAPI, 00-4959-52 Invitrogen, Thermo Fisher Scientific, Waltham, MA, USA).

### 2.5. RNA Extraction of MB Tissue

The 45 sample extractions were performed using the AllPrep RNA FFPE extraction kit (80234 QIAGEN, Hilden, Germany). Five 5 µm thick tissue sections of each FFPE tissue were deparaffinized with QIAGEN’s deparaffinization solution at 56 °C for 10 min and then centrifuged. The technique was developed according to the manufacturer’s instructions. The supernatant was incubated at 80 °C for 15 min. All samples were transferred to RNeasy MinElute Spin Columns (74204, QIAGEN, Hilden, Germany). DNase I was added to the membranes and incubated. The membranes were washed. RNA was eluted with RNAse-free water and quantified using the NanoDrop One C (Thermo Fisher Scientific, Waltham, MA, USA).

### 2.6. Reverse Transcription and Real-Time PCR

Total RNA (1 μg) was reverse transcribed using random hexametric primers and MultiScribeTM Reverse Transcriptase from the High-Capacity cDNA Reverse Transcription Kit (4368814 Applied Biosystems, Thermo Fisher Scientific, MA, USA). TaqMan Fast Advanced Master Mix (Ref. 4368813, Thermo Fisher Scientific, Waltham, MA, USA) was used for real-time PCR. The technique was developed according to the manufacturer’s instructions. The assays analyzed were as follows: *NRP1* (Hs00826128_m1, 4331182, Thermo Fisher Scientific, Waltham, MA, USA) and *18s* (Hs99999901_s1, 4331182 Thermo Fisher Scientific, Waltham, MA, USA) as housekeeping gene. Expression was analyzed by VIC-MGB and FAM-MGB fluorescence in the LightCycler^®^ 480 thermocycler (Roche, Basel, Switzerland). The process comprised 40 cycles with a cutoff at Ct 35 so that only 33 MB samples showed RNA integrity characterized by *18s* amplification. Gene expression was analyzed using the ∆Ct method.

### 2.7. Statistical Analysis

The data are presented as medians and ranges. Statistical dependence between pairs of observations was analyzed using Spearman’s rank correlation coefficient, and comparison between two groups was performed using U-Mann–Whitney. More than two groups were analyzed with the Kruskal–Wallis test and Dunn test as post hoc analysis. Categorical variables were analyzed using the Chi-square test to evaluate associations between groups. For survival analysis, Kaplan–Meier survival curves were generated, and differences between groups were assessed using the log-rank (Mantel–Cox) test. Significance was set at a *p* value of <0.05. The statistical analyzes were performed in the statistical environment R Studio (V.4.3.0). All graphs were created with the program GraphPad Prisma^®^ (V.10.1.2).

## 3. Results

Forty-five MB samples were collected between 2005 and 2022, median age at diagnosis was 7 years (range 1–33), median follow-up time was 12 months (range 1–88). Of the patients, 44.4% (n = 20) were classified as standard risk, and 33.3% (n = 15) had tumor recurrence or progression. MBC histology was present in 51.1% of cases (n = 23), MBDN histology was observed in 24.4% of MB (n = 11), MBEN histology was detected in 6.6% of cases (n = 3), and MBLCA was present in 17.7% of MB (n = 8) (Supplementary Figure S3). MB-WNT was detected in 15.5% of patients (n = 7), MB-SHH in 44.4% (n = 20), MB-No WNT/SHH in 33.3% (n = 15), and 3 cases were classified as MB-NOS (not otherwise specified) group (Table 1).

### 3.1. NRP1 Is Expressed by Tumor-Associated Microglia/Macrophages (TAMs) in MB Tissues Regardless of Their Molecular Subgroup

**Figure 1:**
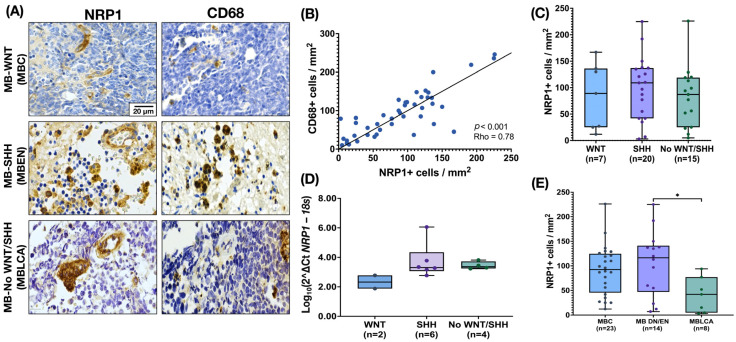
Expression of NRP1 by tumor-associated microglia/macrophages (TAMs) in the MB microenvironment. (**A**) Representative images of MB tissue showing NRP1 staining in cells with glial morphology and CD68 staining in TAMs documented in MB molecular group by IHC (n = 45). (**B**) The density of NRP1 and CD68 cells shows a strong correlation (Spearman correlation coefficient, *p* = 0.0001, Rho = 0.78, IC95% 0.6087 to 0.8710). (**C**) Density of cells NRP1+ by molecular MB subgroup. (**D**) Relative gene expression of *NRP1* in the different molecular MB subgroups (n = 12 with expression of mRNA of *NRP1*—only two WNT samples were available for evaluation). Gene expression was normalized using the *18S* rRNA gene as an internal control. The ΔCT was calculated as CTNRP1mean—CT18Smean, and the relative expression was estimated by the log10 (2^−ΔCT^) transformation. The graph shows the distribution of relative expression values, with individual data points superimposed. The *y*-axis is plotted on a logarithmic scale to reflect the range of relative expression values. (**E**) NRP1 density of cells by MB morphologic patterns. The boxplot in figures (**C**–**E**) shows the median and the range (min–max). Scale bar, 20 μm. * *p* = 0.0287.

**Figure 2:**
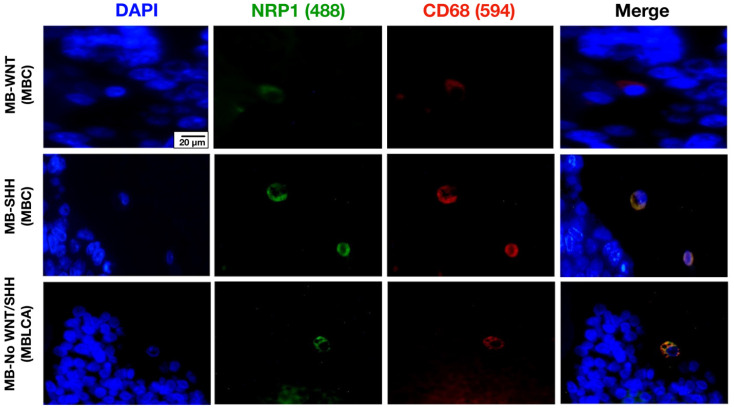
Representative images of tissue from patients diagnosed with MB-WNT (n = 4), MB-SHH (n = 7), and MB-No WNT/No SHH (n = 4), stained for the microglia/macrophage marker CD68 (red), NRP1 (green), and DAPI (blue). Scale bar, 20 μm.

NRP1 staining was analyzed using the IHC technique. NRP1 was detected on occasional tumor cells, but its expression was particularly evident in round cells with glial morphology located between tumor cells and in the endothelium. These round NRP1+ cells showed a high similarity in morphology and distribution width with CD68+ TAMs (Figure 1A). To investigate a possible correlation between these biomarkers, we statistically compared the density of CD68+ and NRP1+ cells and found a strong correlation (*p* < 0.001, Rho = 0.78, IC95% 0.6087 to 0.8710, Figure 1B). Next, we analyzed the density of NRP1+ cells in tumor tissue, which showed a relatively homogeneous distribution across MB molecular subgroups (Figure 1C). This pattern was also consistent when analyzing gene expression levels (Figure 1D). However, comparison by histologic variant revealed a significant increase in MB with nodular histology vs. MBLAC, most of which belonged to the SHH subgroup (Figure 1E). Given the similarity of morphology, distribution, and density of NRP1+ and CD68+ cells, we next investigated the co-expression of both proteins in MB tissue by immunofluorescence. Fluorescence staining is suggestive of co-localization of both proteins in the same cells within the TME (Figure 2).

**Figure 3:**
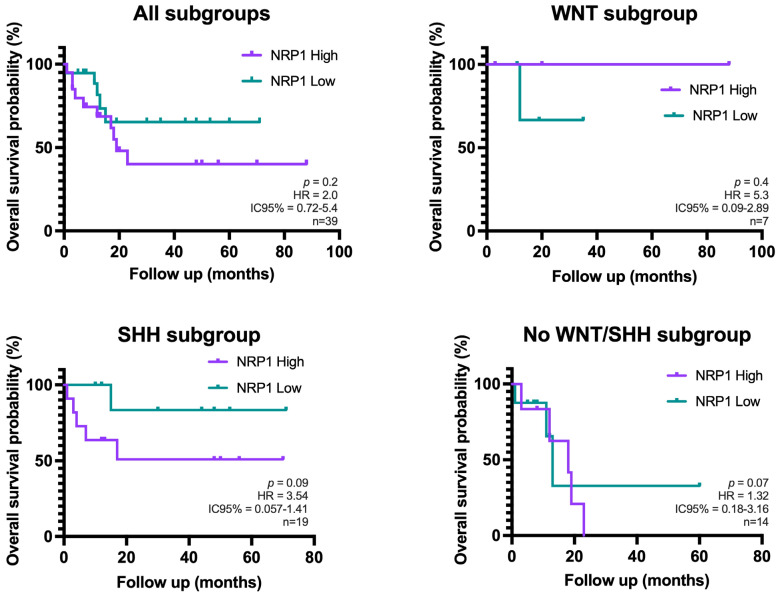
Kaplan–Meier curves showing overall survival of patients with high vs. low NRP1+ cell density in the overall cohort and stratified by molecular subgroups of MB (n = 45). These cases belong to the same cohort analyzed in the previous results. Overall survival was calculated from day of diagnosis to the last follow-up or death. Group comparisons were performed using the log-rank test (Mantel–Cox) (no statistically significant differences were found).

A Kaplan–Meier survival analysis was performed to investigate the relationship between NRP1+ cell density and overall survival (OS). Patients were compared according to their high or low density of NRP1+ cells, with the median NRP1+ cell density serving as the cut-off value. Although patients with low NRP1+ density appear to have a higher probability of cumulative survival in the overall cohort and in the MB-SHH subgroup, this effect did not reach statistical significance (*p* > 0.05) (Figure 3).

### 3.2. Medulloblastoma Tumor Vessels Show Strong NRP1 Expression and Limited Astrocytic Association

**Figure 4:**
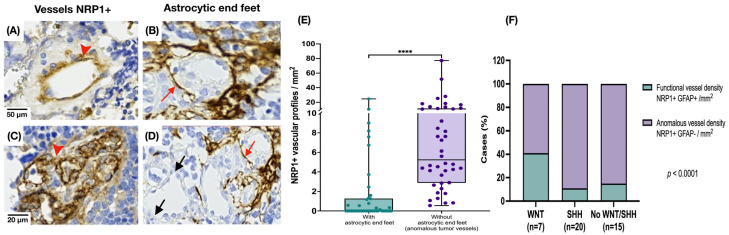
Expression of NRP1 and GFAP in intratumoral blood vessels. (**A**,**C**) NRP1 staining was observed in all endothelia of MB tissue (red arrowhead). (**B**) GFAP immunoreactivity in astrocytic end feet around the vessels (red arrow in (**B**,**D**)). (**D**) Vessels without surrounding GFAP staining (black arrow). (**E**) Higher number of abnormal vessels NRP1+, with no evidence of interaction with astrocytic projections in MB tissue (U-Mann–Whitney, **** *p* = 0.0001). (**F**) The percentage of apparently abnormal vessels NRP1+ GFAP- (without nearby astrocytic projections) and NRP1+ blood vessels preserving an interaction with astrocytes (GFAP+) was calculated by molecular group (chi square test, *p* < 0.0001).

To assess tumor vessel characteristics, we first measured vessel density by quantifying vessel lumina/mm^2^. Vascular density did not differ between the groups. NRP1 expression was detected intensely and diffusely in MB tissue in most tumor vessel endothelium. To investigate the involvement of NRP1 at the BBB, we compared endothelial NRP1 staining with the presence of astrocytic end feet (GFAP+) surrounding or in close contact with tumor vessels. We observed numerous intratumoral vessels with NRP1+ endothelium and without perivascular astrocytic end feet (U-Mann–Whitney, *p* = 0.0001) (Figure 4).

### 3.3. Astrocytes Show a Morphology-Dependent Distribution in the Medulloblastoma

**Figure 5:**
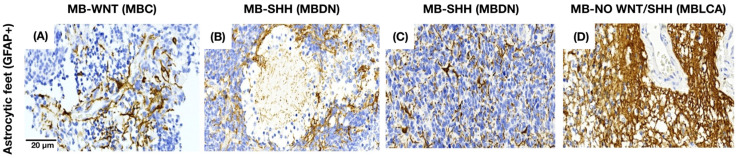
GFAP staining pattern in MB tissue. (**A**) Astrocytes near the endothelium in MBC. (**B**,**C**) Astrocytes between the tumor cells in MBDN and MBEN. (**D**) Astrocytes in both areas in MBLCA.

Astrocytic projections (GFAP+) in MB tissue were identified in three different spatial patterns: adjacent to the endothelium (as part of the BBB), interspersed among tumor cells, or simultaneously in both locations. In MB with a nodular-pattern, astrocytes typically preserve the integrity of the nodular borders and avoid infiltration into the nodular core. In contrast, tumors with classic or anaplastic morphology exhibit a more diffuse astrocytic distribution that is not restricted to specific histologic compartments (Figure 5).

### 3.4. Aberrant GFAP Expression by MB Tumor Cells: Correlation with SHH Subtype and Clinical Outcome

**Figure 6:**
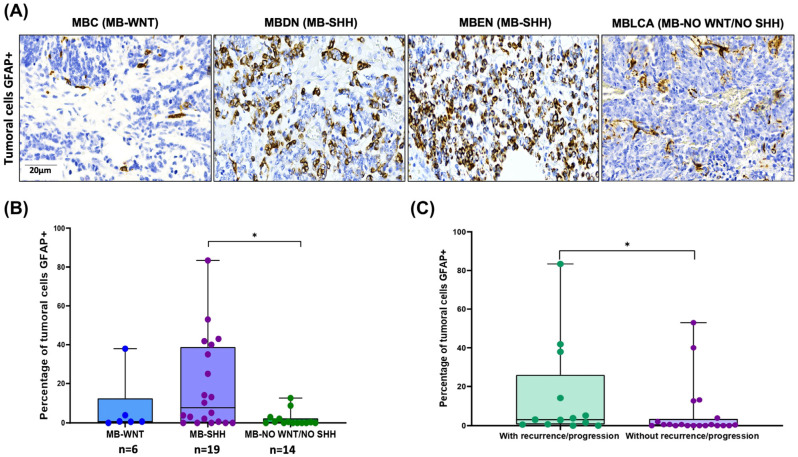
GFAP staining in MB tumor cells and its correlation with clinical features. (**A**) GFAP immunoreactivity in MB tumor cells by histology and molecular group. (**B**) The percentage of tumor cells GFAP+ was higher in MB-SHH molecular group (Kruskal–Wallis, * *p* = 0.02, Dunn test, * *p* = 0.03). (**C**) The percentage of tumor cells GFAP+ was higher in patients with tumor recurrence or progression (U-Mann–Whitney, * *p* = 0.04).

GFAP immunoreactivity was also observed in MB tumor cells (Figure 6A). To investigate the potential clinical significance, we performed a statistical analysis correlating GFAP expression with patient characteristics. A significantly higher percentage of GFAP-positive tumor cells was found in the SHH-subgroup, especially in cases classified as MBDN and MBEN, compared to the WNT and non-WNT/SHH groups (Kruskal–Wallis, *p* = 0.02; Dunn’s post hoc test, *p* = 0.01) (Figure 6B). In addition, cases with tumor recurrence or progression had a higher number of GFAP+ tumor cells (U-Mann–Whitney, *p* = 0.04) (Figure 6C).

## 4. Discussion

In the present study, we report for the first time the expression of NRP1 in tumor-associated microglia/macrophages (TAMs) within the MB microenvironment. NRP1 immunoreactivity was detected in both CD68+ TAMs and endothelial cells using IHC and IF techniques and correlated with *NRP1* gene expression levels in MB tissue. Remarkably, NRP1 protein expression was present in all MB cases, regardless of their molecular subgroup, suggesting that its activation is a conserved feature in MB oncogenesis. This observation is consistent with previous findings showing that NRP1 expression was documented in the immature human cerebellum [13,26], supporting the hypothesis that MB tumor progression may be associated with reactivation of developmental pathways in the cerebellum.

NRP1 is a co-receptor for VEGF and semaphorin signaling that is involved in critical tumor processes such as angiogenesis, metastasis, and tumor progression [37,38]. Its expression may reflect microenvironment dynamics that influence tumor behavior and therapeutic response [23,30,39]. In this study, we showed that NRP1+ non-tumor cells identified in the TME of MB tissues correlate with CD68 expression, indicating high NRP1 expression in TAMs. Expression of NRP1 in tumor-associated macrophages/microglia was previously documented in gliomas and correlates with an immunosuppressive and tumor promoting pathway. Genetic or pharmacological inhibition of NRP1 in glioma TAMs reprograms them towards an antitumor phenotype, by reducing angiogenesis and enhancing lymphocyte infiltration, thus delaying tumor progression [40,41].

In MB, the role of NRP1 has been investigated in both in vitro and in vivo models as well as in human tumor tissue. However, most studies focused on the expression of NRP1 in the tumor cells themselves [12,13], where it is differentially expressed in the various molecular subgroups associated with poor prognosis, as it is involved in promoting metastasis, angiogenesis, and overall tumor progression. On the other hand, the presence and functional status of TAMs in MB have been investigated mainly in the SHH subgroup, where several studies reported higher TAM density within the TME [8,11,14,42], but their functional role remains controversial. While Maximov et al. (2019) showed that TAM infiltration in MB-SHH murine models was associated with decreased tumor growth and increased apoptosis [11], other authors linked the presence of TAM, especially in MB-SHH, to a pro-tumoral phenotype that promotes a unique and permissive tumor environment, and even suggested some of the TAM-specific molecules as potential therapeutic targets [8]. In addition, some studies showed that MB-SHH TAMs exhibit significant functional plasticity and contain both pro- and anti-inflammatory subpopulations [42]. Future studies should aim to characterize the activation state of NRP1+ TAMs in MB to determine whether NRP1 expression influences their pro- or anti-tumor effects and to elucidate their potential role in modulating angiogenesis within the medulloblastoma microenvironment.

In our study, even though NRP1+ cell density did not differ significantly across molecular subgroups, we observed a tendency towards higher density in tumors with nodular histology, majorly in desmoplastic/nodular and extensive nodular subtypes. These variants are frequently associated with MB-SHH with wild-type *TP53*, especially in infants [2,43]. This trend towards greater NRP1 expression in MB-SHH is consistent with previous studies [12,13,44] and is supported by survival data and association with recurrence or progression [12,13]. Kaplan–Meier analysis revealed a trend associating lower NRP1+ cell density with better overall survival when analyzing the complete cohort based on the NRP1+ density (high/low) cells, a trend that remains among SHH tumors. Although these differences were not statistically significant, the observed patterns suggest a possible prognostic role of NRP1 expression in MB. In contrast, NRP1+ cell density appeared to have minimal impact on survival in WNT tumors, which are generally associated with favorable outcomes [4,45]. The lack of statistical significance could be due to the limited sample size, especially when stratified by molecular subgroups. These results contrast with those of De Araujo et al. (2023) where, paradoxically, lower *NRP1* gene expression in human MB-SHH and Group 3 tissues was associated with lower survival, a correlation not observed when analyzing the overall cohort [44]. Nevertheless, the reproducibility of these trends across subgroups supports a biologically meaningful association between NRP1 expression and tumor progression, angiogenesis, and immunomodulation [29,30,44].

Another finding of this study was the expression of NRP1 in the endothelium of tumor vessels, which showed a diffuse pattern and was associated with the formation of aberrant vessels without perivascular astrocytic end feet. These aberrant vessels were observed significantly more frequently in SHH and non-WNT/SHH tumors and less frequently in MB-WNT. Endothelial NRP1 expression has been detected in cultured human cerebral endothelial cells and in the endothelium of human brain and spinal cord tissue in association with neuroinflammatory diseases [46]. This occurs following cerebral endothelial injury, which induces NRP1 expression, impairs BBB integrity and promotes an IFN-γ-mediated inflammatory response [46]. Previous studies documented NRP1 expression in MB tumor cells and its association with angiogenesis and tumor progression [13]. Although MB-WNT tumors are known to exhibit a permeable vasculature with impaired BBB integrity due to specific abnormalities in endothelial and pericyte proteins [33], gliovascular uncoupling has not been previously described in this context or in other MB subgroups.

Although the functional effects on the BBB were not investigated in this study, this phenomenon has been investigated in other tumor types. It was documented in gliomas and metastatic brain tumors [47,48], where disruption of the coupling between astrocytes and vessels leads to altered BBB permeability. In the context of MB, this phenomenon may also reflect the complex immune–glia–tumor interactions within the TME, as another potential source of NRP1 influencing this vascular phenotype is TAMs, as suggested by the present study. NRP1 expression was detected in CD68+ cells, which were significantly more abundant in tumors with nodular histology, which are often associated with alterations in SHH signaling. This raises the possibility that NRP1+/CD68+ cells contribute to the formation, maintenance, or remodeling of abnormal vessels, either through direct cellular interactions or through paracrine signaling within the TME, promoting a pro-tumoral and immunosuppressive environment [40,41].

When examining the GFAP expression, we also found that GFAP was abnormally expressed by tumor cells in all molecular subgroups (with a considerable variation range of 22.8–86.5%), with a significantly higher percentage of expression in SHH-MB cases (*p* = 0.02) and in cases with tumor recurrence or progression (*p* = 0.04). This finding suggests lineage plasticity, which is consistent with studies showing that cerebellar granule cell precursors (CGCP, cells of origin of SHH-MB) can adopt an astrocytic phenotype when exposed to SHH and bone morphogenetic proteins (BMPs) [49]. This partial astrocytic differentiation could reflect aberrant activation of developmental programs within the TME. Such plasticity could underlie the GFAP expression observed in this work and potentially influence tumor behavior and progression. Subsequently, Yao et al. (2020) [9] documented the presence of GFAP+ cells in both mouse models and MB patients. These cells are derived from CGCPs and could promote tumor progression through the secretion of IGF [9]. Interestingly, IGF is known to stimulate cell proliferation in SHH-MB and activate NRP1 expression, possibly through autocrine or paracrine mechanisms [9]. Using proteomic analysis and IHC, Narayan et al. (2020) also reported the expression of GFAP in tumor cells exclusively in SHH-MB tissues and correlated this expression with a favorable prognosis [21]. This finding is in contrast to our results, as we detected GFAP expression in all molecular groups, although it was significantly higher in SHH cases, and it was associated with worse prognosis (disease relapse/progression) in our cohort. Recently, Ghasemi et al. (2024) [14] analyzed a large set of MBEN samples using multimodal single-cell transcriptomics and reported the presence of astrocytic-like malignant cells with GFAP+ phenotypes in SHH-MBEN. They characterized this subset of tumor cells that play a structural role or influence the differentiation and organization of other tumor cells through paracrine signaling [14].

While our study provides novel insights into the expression of NRP1 in MB and its possible association with TAMs, the TME, and tumor vascular proliferation patterns, it is worth noting that our analyses were performed using FFPE tissue, which is prioritized for diagnostic purposes. Therefore, the available material was fully utilized for the experiments presented here, so we could not perform additional evaluations of macrophage polarization markers (such as MRC1, ADM, and cytokine profiles such as IL-6, IL-8, and CCL2), as well as VEGF and other astrocytic markers. In addition, the preservation and storage conditions of FFPE tissue posed some challenges for the RT-qPCR analyses due to lower RNA integrity. Despite these practical limitations, we believe that the molecular and histological findings presented provide a solid foundation for understanding the role of NRP1 in MB. In the future, studies with complementary molecular analyses will be valuable to build on these results and to further elucidate the immunological features and vascular interactions that shape the microenvironment of MB.

## 5. Conclusions

Overall, our study provides the first evidence for the expression of NRP1 by tumor-associated microglia/macrophages (TAMs) and endothelial cells in the microenvironment of medulloblastoma tumors. Furthermore, we observed the formation of abnormal vessels with endothelial NRP1 expression but without astrocytic projections, a pattern that appears to be more pronounced in SHH-MB and non-WNT/SHH groups. We also found GFAP expression in MB tumor cells, which was significantly higher in the SHH-MB subgroup and appeared to be associated with worse outcomes, such as recurrence or progression. Taken together, these results suggest that NRP1 expression in TAMs and endothelial cells may play a role in shaping a microenvironment conductive to disease progression and abnormal tumoral vascular architecture, possibly contributing to BBB dysfunction through reduced astrocytic interactions. However, it is important to interpret these results with caution, as they need to be confirmed by other methods and in larger samples or in controlled experiments. Nevertheless, we believe that these observations provide the basis for future research to investigate the context-dependent role of NRP1 and TAMs in the medulloblastoma microenvironment and its potential impact on different molecular subgroups.

## Figures and Tables

**Table 1:** Clinical and sociodemographic characteristics of the patients.

Characteristics	n/Median	Percentage/Range
Sex (Male/Female)	33/12	73.33/26.66
Age (median, range) Follow-up time (months)	7 12	1–33 1–88
Risk stratification		
Standard	20	44.44
High	17	37.77
Not available	8	17.77
Recurrence/Progression	15	33.33
Death	15	33.33
Histological subtypes		
MBC	23	51.11
MBDN	11	24.44
MBEN	3	6.66
MBLCA	8	17.77
Molecular subtypes		
MB-WNT	7	15.55
MB-SHH	20	44.44
MB-No WNT/No SHH	15	33.33
MB-NOS	3	6.66

MB, medulloblastoma; MBC, MB classic; MBDN, MB desmoplastic/nodular; MBEN, MB extensive nodular; MBLCA, MB large cell/anaplastic; MB-WNT, MB WNT-activated; MB-SHH, MB Sonic hedgehog-activated; MB-NOS, MB not otherwise specified.

## Data Availability

The data presented in this study are available on request from the corresponding author.

## Supplementary Materials

The following supporting information can be downloaded at: https://www.mdpi.com/article/10.3390/cancers17152417/s1, Figure S1: IHC positive and negative controls; Figure S2: Cell counting strategy; Figure S3: Representative photomicrographs of MB histological variants.

## Abbreviations

The following abbreviations are used in this manuscript:

BBB	Blood–brain barrier
GFAP	Glial fibrillary acidic protein
MB	Medulloblastoma
MBC	Medulloblastoma, classic
MBDN	Medulloblastoma, desmoplastic/nodular
MBEN	Medulloblastoma, extensive nodular
MBLCA	Medulloblastoma, large cell/anaplastic
NRP1	Neuropilin-1
PIGF	Placental growth factor
SHH	Sonic hedgehog
TAMs	Tumor-associated microglia/macrophages
TGF-β	Transforming growth factor-beta
TME	Tumor microenvironment
VEGF	Vascular endothelial growth factor
WNT	Wnt/Wingless
