# Supplementary material for: NRP1 and GFAP Expression in the Medulloblastoma Microenvironment: Implications for Angiogenesis and Tumor Progression
Source: Cancers (Basel). 2025 Jul 22;17(15):2417. doi: 10.3390/cancers17152417 (PMC12345964; doi:10.3390/cancers17152417)
Supplement: Supplementary file 1 [file cancers-17-02417-s001.zip › cancers-3730136-supplementary.pptx]

## Slide 1
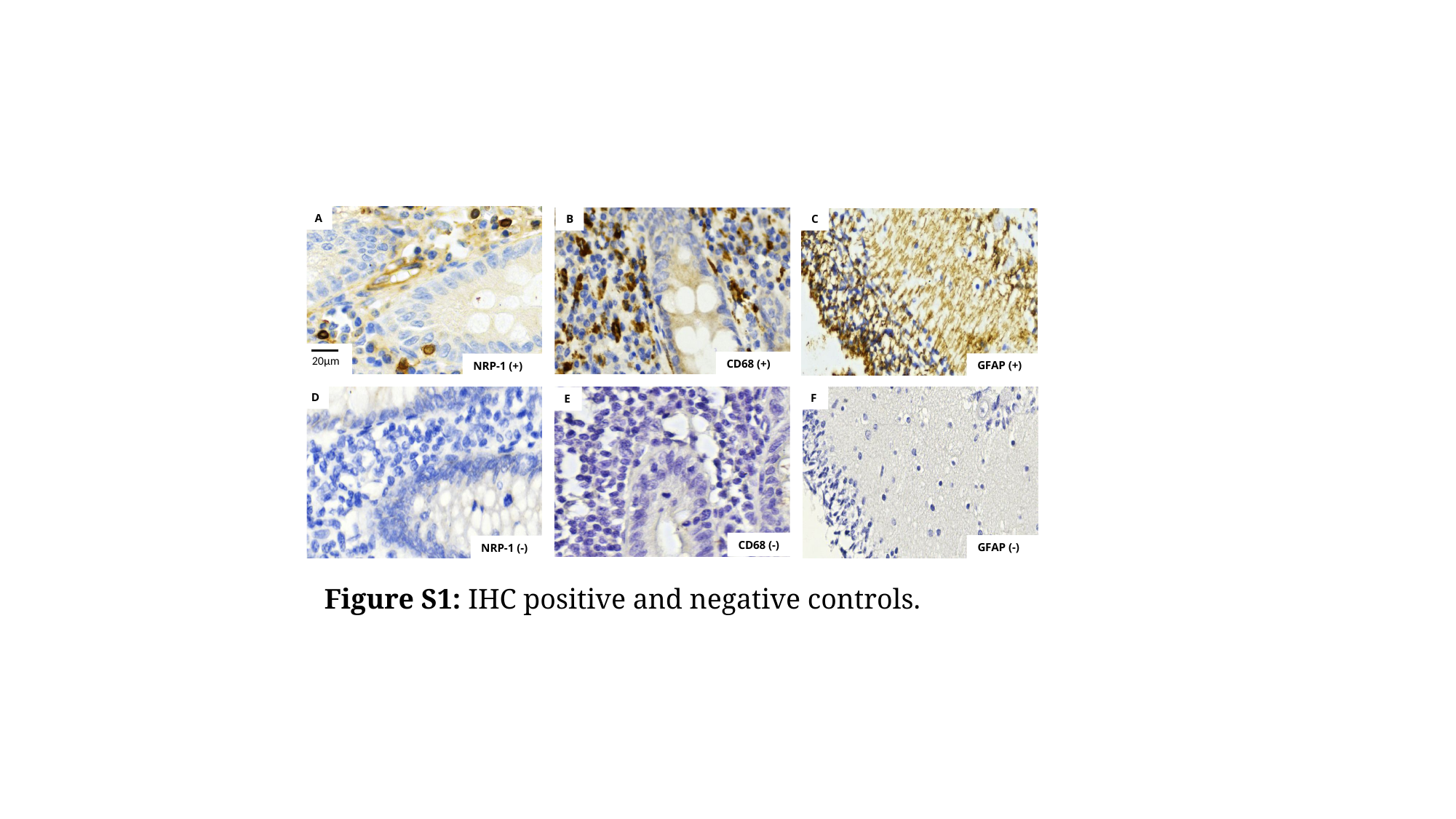

A
C
B
20µm
CD68 (+)
GFAP (+)
NRP-1 (+)
D
F
E
CD68 (-)
GFAP (-)
NRP-1 (-)
Figure S1: IHC positive and negative controls.

## Slide 2
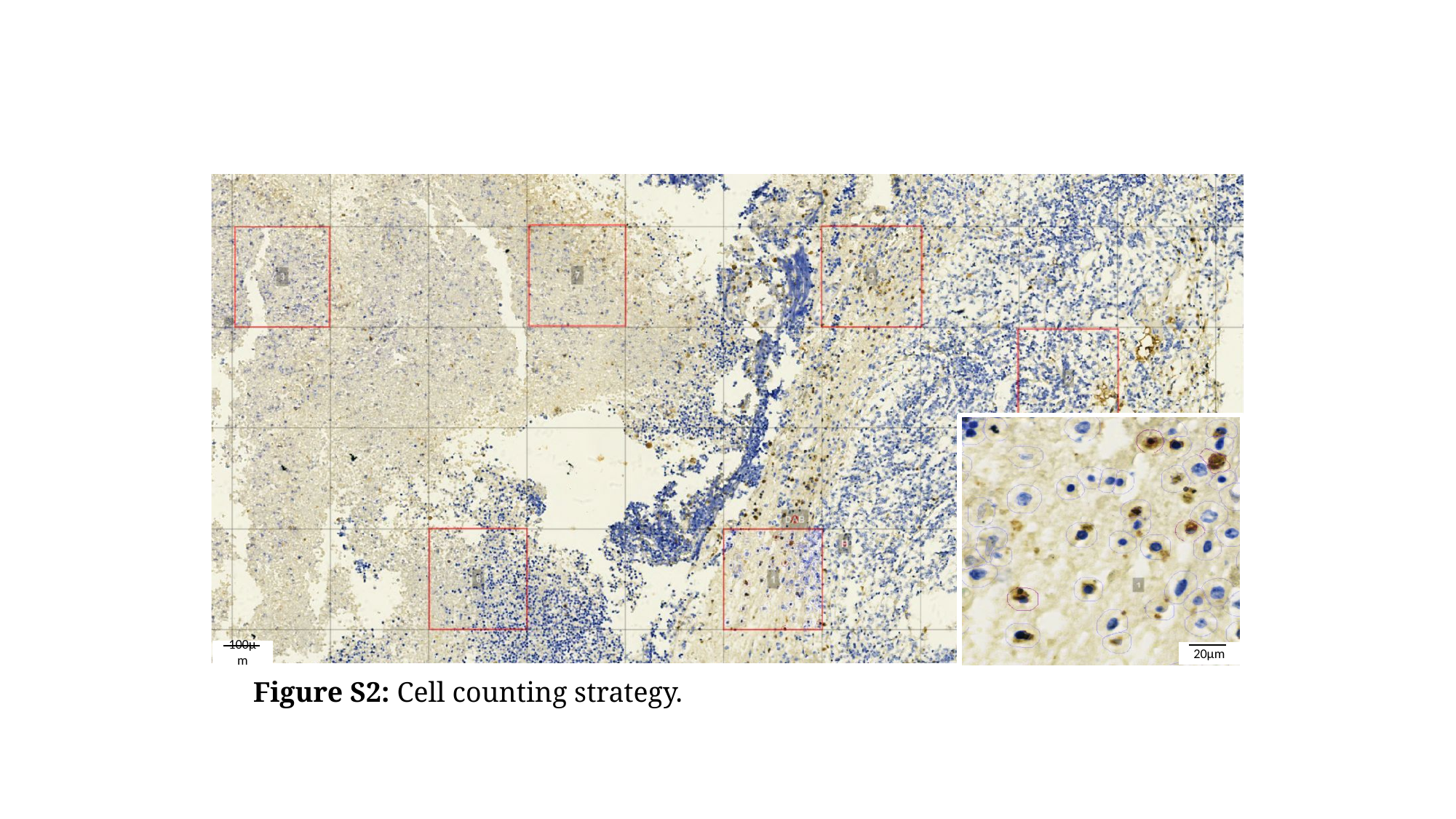

100µm
20µm
Figure S2: Cell counting strategy.

## Slide 3
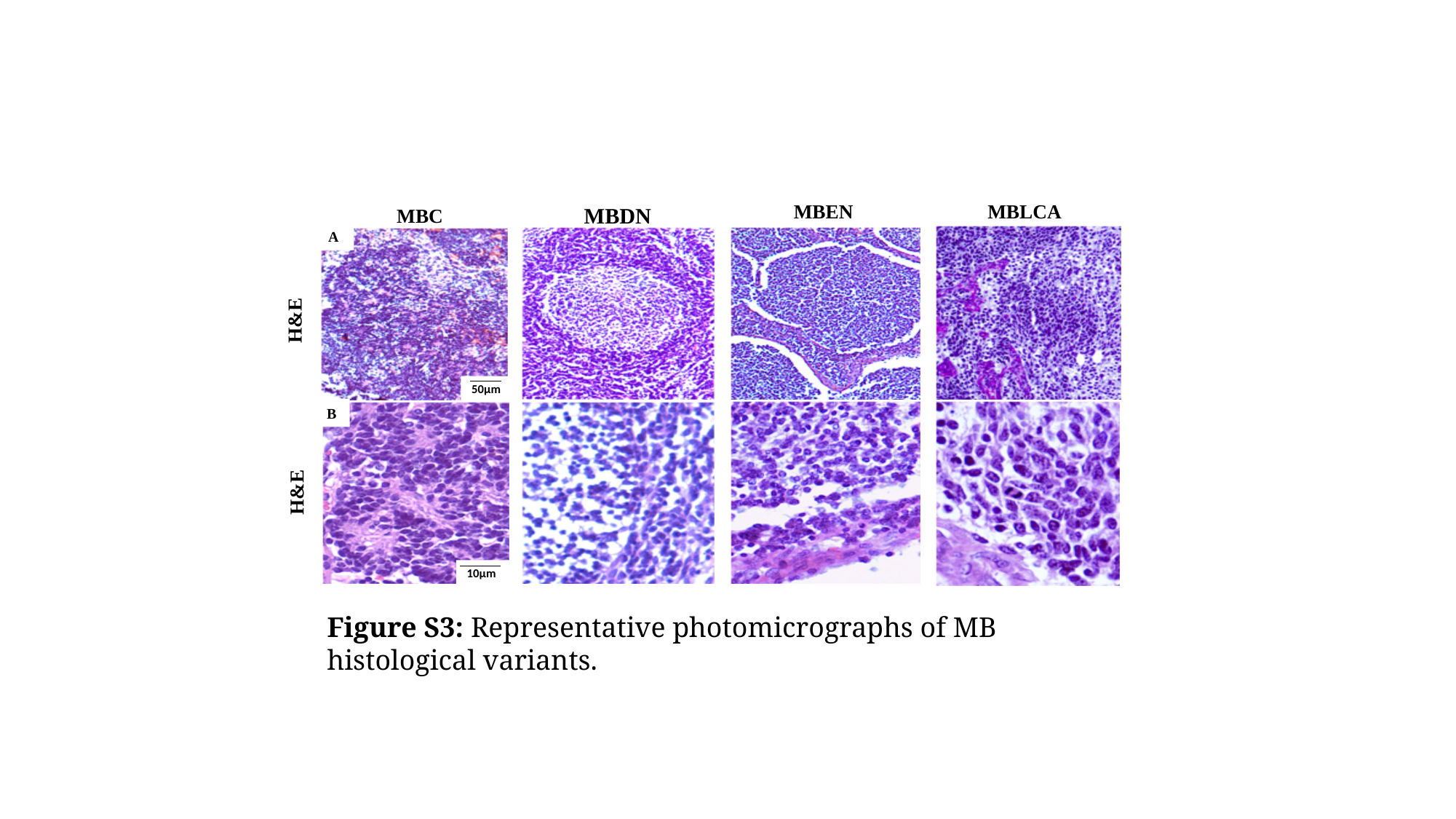

H&E
H&E
A
MBC
MBDN
MBEN
MBLCA
B
10μm
50μm
Figure S3: Representative photomicrographs of MB histological variants.
